# Multi-omics analysis reveals the effects of *Codonopsis pilosula polysaccharides* on the skeletal muscle development of Sansui ducks during the embryonic stage

**DOI:** 10.1016/j.psj.2026.107595

**Published:** 2026-08-09

**Authors:** Ai Liu, Yifu Rao, Yong Yue, Jinjin Zhu, Jiying Wen, Biqiong Yao, Yongcai Zhu, Surintorn Boonanuntan, Shenglin Yang

**Affiliations:** a Key Laboratory of Animal Genetics, Breeding and Reproduction in the Plateau Mountainous Region, Ministry of Education, Guizhou University, Guiyang, 550025, China; b College of Animal Science, Guizhou University, Guiyang, 550025, China; c School of Materials Science and Engineering, Guizhou Minzu University, Guiyang, 550025, China; d Agricultural and Rural Bureau of Sansui County, Sansui County 556500, China; e School of Animal Production and Innovation, Suranaree University of Technology, Nakhon Rachasima, 30000, Thailand

**Keywords:** *Codonopsis pilosula polysaccharides* (CPPS), Skeletal muscle, Multi-omics, mTOR signaling pathway, Glycerophospholipid metabolism

## Abstract

The present study aimed to explore the difference and its underlying molecular mechanism of in ovo feeding (IOF) with *Codonopsis pilosula polysaccharides* (CPPS) on skeletal muscle in ducks. A total of 120 fertile eggs were randomly distributed into two treatment groups, with 6 replicates per group and 10 eggs per replicate. IOF was performed at incubation day 13 (I13). The control group was injected with 0.2 mL 0.75% sterile saline, while the CPPS group received 0.2 mL CPPS solution at a dose of 3 mg per egg. Breast muscle and leg muscle tissue samples were collected at I24 for multi-omics (transcriptomics, metabolomics and proteomics) analyses. In breast muscle, a total of 1301 differentially expressed genes (DEGs) were identified (497 upregulated and 804 downregulated). Genes involved in muscle development, such as creatine kinase, mitochondrial 2 (*CKMT2*, ENSAPLG00020008721) and G-protein signaling 4(*RGS4*, ENSAPLG00020014659), were significantly upregulated. For proteomics, 89 different expressed proteins (DEPs) were identified (44 upregulated and 45 downregulated), among which, Eukaryotic translation initiation factor 4E type 2 (*eIF4E2*, A0A8B9SQK7), myomesin 3 (A0A8B9ZBZ5), myogenic factor 6 (A0A8B9SGC1), myosin heavy chain and other myogenic-related proteins (*MyHC*, A0A8B9ZL15) related to muscle synthesis were markedly increased. Metabolomic analysis identified 1647 differential metabolites (DEMs), including 43 upregulated and 22 downregulated, with leucine being significantly decreased. Multi-omics integration revealed that these differential molecules were collectively enriched in the mTOR signaling pathway and glycerophospholipid metabolism pathway. These coordinated regulatory effects synergistically promoted breast muscle fiber development, enhanced energy metabolism, alleviated stress induced injury and ultimately improved skeletal muscle growth potential and meat quality. While in the leg muscles, 1195 DEGs were identified (487 upregulated and 708 downregulated), among which the expression of transcription factor 15 (*TCF15*, ENSAPLG00020006012), a gene associated with muscle development, was significantly upregulated. Proteomic analysis revealed 47 DEPs (15 upregulated and 32 downregulated), including phosphorus receptor protein (A0A8B9TCV3, *PLN*), mitofusin 1(A0A8B9TRP9, *MFN1*), titin(A0A8B9ZFJ8), which are closely related to muscle development and energy metabolism. Metabolomic analysis identified 1647 DEMs were identified (66 upregulated and 40 downregulated), with 5-Hydroxy-L-tryptophan (*5-HTP*), the precursor of serotonin (*5-HT*), significantly upregulated, providing substrates and energy for muscle development. Although no shared enriched pathway existed across the three omics in leg muscle, the differential molecules collectively suggest that IOF with CPPS supports energy supply for cell proliferation and creates a favorable environment for myogenesis. These findings help to elucidate the molecular mechanisms by which CPPS regulates embryonic skeletal muscle development. Moreover, the candidate genes, proteins and metabolites identified in this study may serve as valuable biomarkers and provide a potential nutritional strategy for improving meat quality traits in duck breeding.

## Introduction

In modern animal husbandry, skeletal muscle is regarded as the most economically valuable part in livestock and poultry. As the largest muscle tissue type, the growth and development of skeletal muscle directly determine duck meat quality, which represents one of the most critical economic traits in poultry genetic breeding (Vida et al., 2023). Accordingly, enhancing muscle growth rate and meat quality has become a major objective in animal production. Prenatal myogenesis is a sophisticated biological process, including the proliferation and differentiation of myogenic precursor cells, the fusion of differentiated myoblasts into multinucleated myotubes, and the subsequent formation of mature muscle fibers (Stewart and Rittweger, 2006; Grand and Rudnicki, 2007). In poultry, the hyperplasia process of muscle fiber is nearly complete during time of hatch. After hatching, the muscle fibers grow mainly via hyperthrophy (Velleman, 2007; Wang et al., 2022). Therefore, optimizing embryonic skeletal muscle development, especially increasing the number of myofibers or improving their initial quality has become a promising strategy to enhance the subsequent growth performance and meat quality of ducks.

Studies on duck skeletal muscle development mainly focus on molecular genetic regulation and dietary intervention, with most experiments performed on growing ducklings and laying female ducks. (Li et al., 2022; Lin et al., 2022). As a critical window for early duck development, the incubation period allows exogenous nutrient delivery via in ovo feeding (IOF) to exert persistent regulatory effects on duck growth (Kucharska-Gaca et al., 2017). Compared with dietary supplementation, the IOF has emerged as an efficient strategy to deliver bioactive substances directly into embryos.

IOF with various nutrients can promote the development of poultry skeletal muscles in multiple aspects, such as cell proliferation, energy supply, regulation of proliferation genes, and improvement of growth performance (Sun et al., 2026). Recent studies have shown that IOF with nutrients can regulate the skeletal muscle growth and development, hatching performance and intestinal microbial activity (Das et al., 2021). IOF with folic acid increased the hatchability and decreased the embryo mortality, pipped eggs and deformed chicks in Chinese white ducks (Hussian et al., 2019). In geese, IOF with methionine and/or disaccharide enhanced the expression of myogenic factor-5 (*Myf-5*) from breast muscle and sodium/glucose cotransporter protein-1 (*SGLT-1*) from jejunum, also improved the relative weight of breast muscle and small intestine, diameter of myofiber, length of small intestine, jejunal villus width (Dang et al., 2022).

In recent years, plant-derived polysaccharides have attracted extensive attention due to their ability to improve poultry muscle production performance by regulating antioxidant enzyme activity and inhibiting inflammatory responses. Regarding maternal nutritional regulation, dietary supplementation of 30, 60 mg/kg mulberry-leaf flavonoids (MLF) in breeder hens upregulated the expression of *Myf5* and activated the BMPR2/p-Smad1/5/9 and mTOR signaling pathways in offspring during the embryonic period, thereby promoting muscle fiber development and hypertrophy (Huang et al., 2024). This maternal effect persisted until 14 days of age in chicks. In broilers, dietary supplementation with *Chinese yam* polysaccharides (CYP) significantly 500 mg/kg upregulated the expression of *MyoD* and MyoG in breast muscle but no significant difference in leg muscle, and downregulated *MSTN* expression, indicating that CYP could be used as a potential feed additive to promote the development of muscle tissues in broilers(Deng et al., 2022). Dietary supplementation with the polysaccharide of *Millettia speciosa Champ. ex* Benth (MSCP) and glycyrrhiza polysaccharides have been reported to upregulate muscle growth-related genes in chickens, validating their role as promising botanical feed additives that enhance growth performance and improve meat quality (Li et al., 2024; Liu et al., 2025).

*Codonopsis pilosula* (Franch.) *Nannf.,* is a well-known edible and medicinal plant with a long history of application. The roots of *C.pilosula* mainly contains polysaccharides, lignans, polyyne and alkaloids. As one of the major bioactive components, *C.pilosula* polysaccharides (CPPS) exhibit immunomodulatory(Gong et al., 2022), antioxidant(Zou et al., 2021), anti-inflammatory(Zhou et al., 2024), and anti-fatigue effects(Xie et al., 2020). Furthermore, selenization modification of CPPS have attracted considerable attention due to its improved bioavailability and utilization (Luan et al., 2021). The total polysaccharide content in *C. pilosula* can reach 6%. CPPS administered alone or in combination has been reported to promote growth in animals. Dietary CPPS supplementation can enrich beneficial bacteria, modulate gut microbial structure, improve intestinal barrier integrity, and strengthen immune function in Hu sheep (Zhao et al., 2025). Meanwhile, in broilers, CPPS has been demonstrated to enhance antioxidant status and elevate anti-inflammatory cytokine concentrations in serum and liver tissue (Liu et al., 2023). Although CPPS have been reported to improve antioxidant function and growth performance in livestock and poultry, their direct regulatory effects on skeletal muscle development during the embryonic stage of ducks remain unexplored.

In addition, multi-omics integration analysis has been widely employed to explore the underlying molecular mechanisms of complex traits and diseases. For example, a combined metabolomic and transcriptomic strategy has been utilized to identify the differential metabolites and key genes associated with meat quality in Taihe black-bone silky fowl at different age (Huang et al., 2024). Furthermore, integrated transcriptomic and untargeted metabolomic analyses have been applied in Pekin ducks to elucidate the potential molecular mechanisms underlying skeletal muscle development (Hu et al., 2023).

Based on the above backgrounds, the present study aimed to investigate the effects of in ovo feeding (IOF) of CPPS on skeletal muscle development in duck embryos. An integrated multi-omics strategy combining transcriptomics, proteomics, and metabolomics were used to systematically characterize changes in gene expression, protein abundance, and metabolic profiles in duck embryonic skeletal muscle. The findings of this study are expected to provide novel theoretical insights into the regulatory roles of plant polysaccharides in avian embryonic myogenesis and offer a promising practical strategy for improving poultry muscle production potential.

## Materials and methods

### Ethics statement

All animal experiment protocols were approved by the The Animal Welfare Committee of Guizhou University (Guiyang, Guizhou, China) (EAE-GZU-2025-E006).

### Experimental design

#### In ovo feeding test

A total of 120 fertilized Sansui duck eggs weighing on average 68 ± 0.75 g were purchased from Guizhou Sansui Duck Industry Operation and Development (Group) Co., Ltd., Guizhou, China. Eggs were incubated in the College of Animal Science duck hatchery with standard conditions (37.8°C and 60% relative humidity). On the incubation day 9 (I9), the unfertilized and nonviable eggs were removed. Eggs were divided into two groups: the control group (injected 0.2 mL 0.85% saline solution) and the IOF CPPS group (injection volume of 0.2 mL with amount per embryo of 3 mg CPPS in 0.85% saline solution). On I13, yolk sac injection by IOF was performed. On I24, all birds in two treatments were enthanized by cervical dislocation, the breast and leg muscle were collected. All samples were snap frozen in liquid nitrogen and stored at −80 for subsequent analysis.

#### Transcriptomics

Six breast muscle samples and six leg muscle samples from each group were used to perform transcriptomics analysis. RNA sequencing was carried out on an Illumina Novaseq™ X Plus (LC-Bio Technology CO., Ltd., Hangzhou, China) platform using protocols for 2 × 150 bp paired-end sequencing (PE150) with the aim of obtaining 153.96 G raw data for mRNA. DEGs between 2 biological comparisons were identified by DESeq 2 software. The identified genes with *P* < 0.05 and |log_2_FC | ≥ 1 were considered to be differentially expressed genes (DEGs). Gene ontology (GO) and Kyto Encyclopedia of Genes and Genomes (KEGG) pathway enrichment analyses of DEGs were performed.

#### RNA-seq Validation by qRT-PCR

Ten DEGs (7 genes of breast and 3 genes of leg) in the skeletal muscle growth were selected for qRT-PCR analyses. Beta-actin (*β*-actin) was used as an internal marker to normalize the expression levels. Total RNA from breast and leg muscle samples was obtained using E.Z.N.A. Total RNA Kit Ⅰ (Omega Bio-tek, Norcross, GA, USA). The total RNAs (∼1 µg) was reversed transcribed to cDNA using kits (Tiangen Biotech (Beijing) Co., Ltd., China). Real-time PCR was carried out on an CFX96 Touch Real-Time PCR Detection System (Bio-Rad, USA) using a kit (Genstar, A301). Each sample was analyzed in triplicate and relative quantification of gene expression was performed using 2^−ΔΔCt^ method. The primer sequences can be found in Table 1. Statistical significance of data was assessed using one way analysis of variance (ANOVA) followed by Duncan’s test and Tukey’s test. Data were considered significant at *P* < 0.05 and extremely significant at *P* < 0.01, as well as *P* value between 0.05 and 0.1 were considered as a trend.

**Table 1:** Primer sequences for real-time quantitative PCR analysis.

Gene name	Genebank number	Primer sequence (5′ to 3′)	Product size (bp)
breast			
CKMT2	XM_038170754.2	CCGAGCAGTGTTTGAGGG	215
		ATGGGAATAACGAGTTGGTG	
RGS4	XM_038183342.2	ATCCTGACACCGTAGC	147
		GGTAGTGGTCCTATCCC	
Histone H2A-Ⅳ	XM_005012173.6	ACGAGGAGCTCAACAAGCTG	104
		CTTGTGGCTGTCGGTCTTCT	
NFKBIA	XM_027458615.3	CTCCAGCATACAGGAACAGC	177
		CACCAGCCAAACCACAAA	
DKK2	XM_005019820.6	CTAAATCACGGCTTCACG	116
		GCAGTCCTTGTTTCCCAC	
DDIT4	XM_027460245.3	GAGTAGGGTGTAGGTGGACG	177
		CACAAAGAATTAACCGGGAG	
SCD1	KF185111.1	CCATCGGTCCTACAAAGC	178
		GCCAATGTGGGAGAAGAAA	
leg			
TCIF15	XM_027473326.3	GACCGCAAGCTGTCCAAA	128
		GCACCGTAGATGGCACTGAA	
MRP-126	XM_038168061.2	GCTCGTGAACTACCTGAA	106
		CATCACCTCGCCAAAA	
LECT2	XM_027468633.3	TGCCATCTCCAGTGTTGC	132
		CCTTTGTGCGTCCTCCTT	
β-actin	EF667345.1	TGGCATCCACGAAACTAC	215
		AGCCTCCAATCCAGACAG	

#### Proteomics

Each group contained three biological replicates. Nano-LC-MS/MS based TMT quantitative proteomics analysis mainly included protein extraction, quantification and quality inspection, tryptic digestion, desalting, TMT labelling, peptide separation, mass spectrometry detection, database identification, and bioinformatics analysis. The Uniprot Anas platyrhynchos platyrhynchos database was employed for this analysis. The differential proteins (DEPs) were screened using *P* < 0.05 and fold change (FC) ≥ 1.2 or FC ≤ 0.83. GO and KEGG pathway enrichment analysis of DEPs were performed.

#### Metabonomics

Six breast muscle samples and six leg muscle samples from each group were used to conduct metabonomics analysis. Metabonomics analysis was performed with a UPLC‒ESI‒HR-MS/MS system (UPLC, Thermo Vanquish Flex system, MS, Thermo Orbitrap Exploris 120) equipped with an ESI ion source, and metabolites were detected in both positive and negative ion modes with full scan and DDA acquisition modes. The differential metabolites (DEMs) were screened using *P* < 0.05 and fold change (FC) ≥ 1.2 or FC ≤ 0.83, and VIP ≥ 1.

#### Conjoint analysis

Differentially expressed genes (DEGs), proteins (DEPs), and metabolites (DEMs) in breast and leg muscle were screened. An integrated database was established in Excel, and cloud-based bioinformatic tools were employed for pathway enrichment analysis and correlation clustering. The regulatory network among DEGs, DEPs, and DEMs was further characterized. Comparisons between two groups were done with T-test.

## Results

### Descriptive statistics of RNA-seq data

The statistical information of RNA-seq data was presented in the Table 2. From Table 2, in can be seen that the sequencing of breast and leg muscle tissue produced an average about 43.00 million raw reads per sample, with an average base of 6.45 G. After filtering, the number of valid reads was about average 40.85 million raw reads per sample, with an average lase of 6.12 G, and the percentage of valid reads was all larger than 94.09%. The Q20 for each sample was 100%, and Q30 for each sample ranged from 99.07 to 99.18%.

**Table 2:** Statistical information of RNA seq data.

Sample	Raw Data		Valid Data		Valid ratio(%)	Q20%	Q30%	GC content%
	Read	Base	Read	Base				
CPPS_1B	38065350	5.71G	36060506	5.41G	94.73	100.00	99.11	49.50
CPPS_2B	42582544	6.39G	40219542	6.03G	94.45	100.00	99.07	49.50
CPPS_3B	44417088	6.66G	41935080	6.29G	94.41	100.00	99.14	49.00
CPPS_4B	40696698	6.10G	38594304	5.79G	94.83	100.00	99.16	49.00
CPPS_5B	39240518	5.89G	37192018	5.58G	94.78	100.00	99.15	49.50
CPPS_6B	46294400	6.94G	43722466	6.56G	94.44	100.00	99.08	49.00
Control_1B	41298524	6.19G	39038714	5.86G	94.53	100.00	99.12	49.50
Control_2B	42542564	6.38G	40210560	6.03G	94.52	100.00	99.10	49.00
Control_3B	38975850	5.85G	36673468	5.50G	94.09	100.00	99.08	49.00
Control_4B	48502106	7.28G	45973544	6.90G	94.79	100.00	99.11	49.50
Control_5B	46895478	7.03G	44335172	6.65G	94.54	100.00	99.09	49.50
Control_6B	41824528	6.27G	39666146	5.95G	94.84	100.00	99.13	49.50
CPPS_1L	43163930	6.47G	41141730	6.17G	95.32	100.00	99.18	50.00
CPPS_2L	43501740	6.53G	41587200	6.24G	95.60	100.00	99.15	50.00
CPPS_3L	43045824	6.46G	41014312	6.15G	95.28	100.00	99.17	49.50
CPPS_4L	45923748	6.89G	43738896	6.56G	95.24	100.00	99.18	49.50
CPPS_5L	39106536	5.87G	37297338	5.59G	95.37	100.00	99.17	50.00
CPPS_6L	40360538	6.05G	38530166	5.78G	95.46	100.00	99.13	50.00
Control_1L	41298524	6.19G	39038714	5.86G	94.53	100.00	99.12	49.50
Control_2L	42542564	6.38G	40210560	6.03G	94.52	100.00	99.10	49.00
Control_3L	38975850	5.85G	36673468	5.50G	94.09	100.00	99.08	49.00
Control_4L	48502106	7.28G	45973544	6.90G	94.79	100.00	99.11	49.50
Control_5L	46895478	7.03G	44335172	6.65G	94.54	100.00	99.09	49.50
Control_6L	41824528	6.27G	39666146	5.95G	94.84	100.00	99.13	49.50

### Differential expression genes (DEGs) analysis

**Fig. 1:**
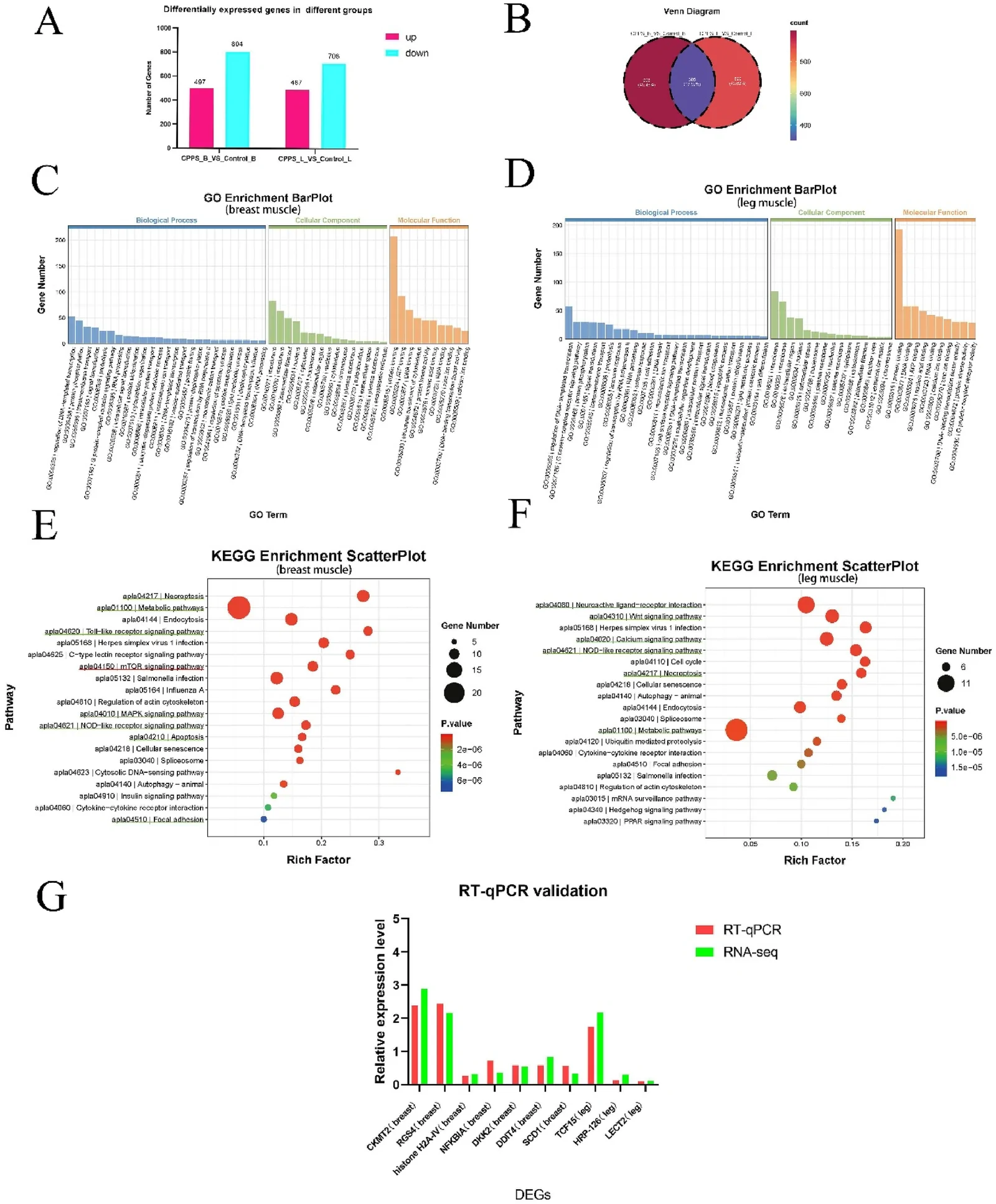
(A) Number of upregulated and downregulated DEGs between different groups. (B) Venn diagram of DEGs among the two comparison groups. (C-D) GO enrichment analysis of DEGs in skeletal muscle. (C) for CPPS_B VS Control_B, (D) for CPPS_L VS Control_L. (E-F) KEGG pathways of DEGs enrichment in skeletal muscle. (E) for CPPS_B VS Control_B, (F) for CPPS_L VS Control_L. (G) RT-qPCR validation for the DEGs.

Through differential expression analysis, 1301 DEGs were detected for CPPS_B VS CON_B with 497 up-regulated and 804 down-regulated, 1195 DEGs were detected for CPPS_L VS CON_L with 487 upregulated and 708 downregulated. The CPPS_B group showed significant upregulation of regulator of G-protein signaling 4 (*RGS4*), creatine kinase, mitochondrial 2 (*CKMT2*), along with significant downregulation of histone H2A-IV, nuclear factor of kappa light polypeptide gene enhancer in B-cells inhibitor alpha (*NFKBIA*), dickkopf Wnt signaling pathway inhibitor 2 (*DKK2*), DNA-damage-inducible transcript 4 (*DDIT4)*, and stearoyl-CoA desaturase 1 (*SCD1*). In the CPPS_L group, the expression of transcription factor 15 (*TCF15*) was significantly upregulated, while that of myeloid-related protein 126 (*MRP-126*, or *SA100A12*), leukocyte cell-derived chemotaxin 2 (*LECT2)*, was significantly downregulated (Fig. 1A, 1B).

### GO and KEGG analysis

To gain further insight into the developmental processes differing of skeletal muscle between 2 groups, analysis of GO terms and KEGG pathways were performed and the top 20 GO biological process and KEGG pathways were selected to co-enriched factor figures (Fig. 1C to F).

For the CPPS_B VS CON_B, the GO terms of intermediate filament, structural constituent of cytoskeleton, protein binding and protein kinase activity were significantly enriched (*P* < 0.05). Protein binding participates in the regulation of myoblast proliferation and differentiation, thereby affecting skeletal muscle development. KEGG pathway analysis showed that DEGs were mainly enriched in Metabolic pathways, Necroptosis, Toll-like receptor signaling pathway, mTOR signaling pathway, NOD-like receptor signaling pathway, MAPK signaling pathway, Apoptosis, and Focal adhesion (*P* < 0.05). MAPK signaling pathway, Wnt signaling pathway were related to cell proliferation and differentiation.

For the CPPS_L VS CON_L, the GO terms of protein biding, extracellular region, defense response, immune response, cell differentiation were significantly enriched (*P* < 0.05). KEGG pathway analysis showed that DEGs were mainly enriched in Metabolic pathways, Neuroactive ligand-receptor interaction, Wnt signaling pathway, NOD-like receptor signaling pathway, Necroptosis, and Calcium signaling pathway (*P* < 0.05). Among them, Metabolic pathways, Necroptosis, NOD-like receptor signaling pathway, Wnt signaling pathway were the shared pathways enriched by DEGs in CPPS_B VS CON_B.

### qRT-PCR validation of RNA-seq results

The results showed that the expression trends determined by qRT-PCR were consistent with the RNA-Seq results. Compared with CON group in the breast muscle, CPPS_B group exhibited up-regulation of *CKMT2, RGS4* (*P* < 0.05) and down-regulation of histone *H2A-IV, NFKBA, DKK2, DDIT4, SCD1*(*P* < 0.05). Compared with CON group in the leg muscle, CPPS_L group exhibited up-regulation of *TCF15*(*P* < 0.05), and down-regulation of *MRP-126* and *LECT2* (*P* < 0.05) (Fig. 1G).

### Identification and comparison of DEPs

**Fig. 2:**
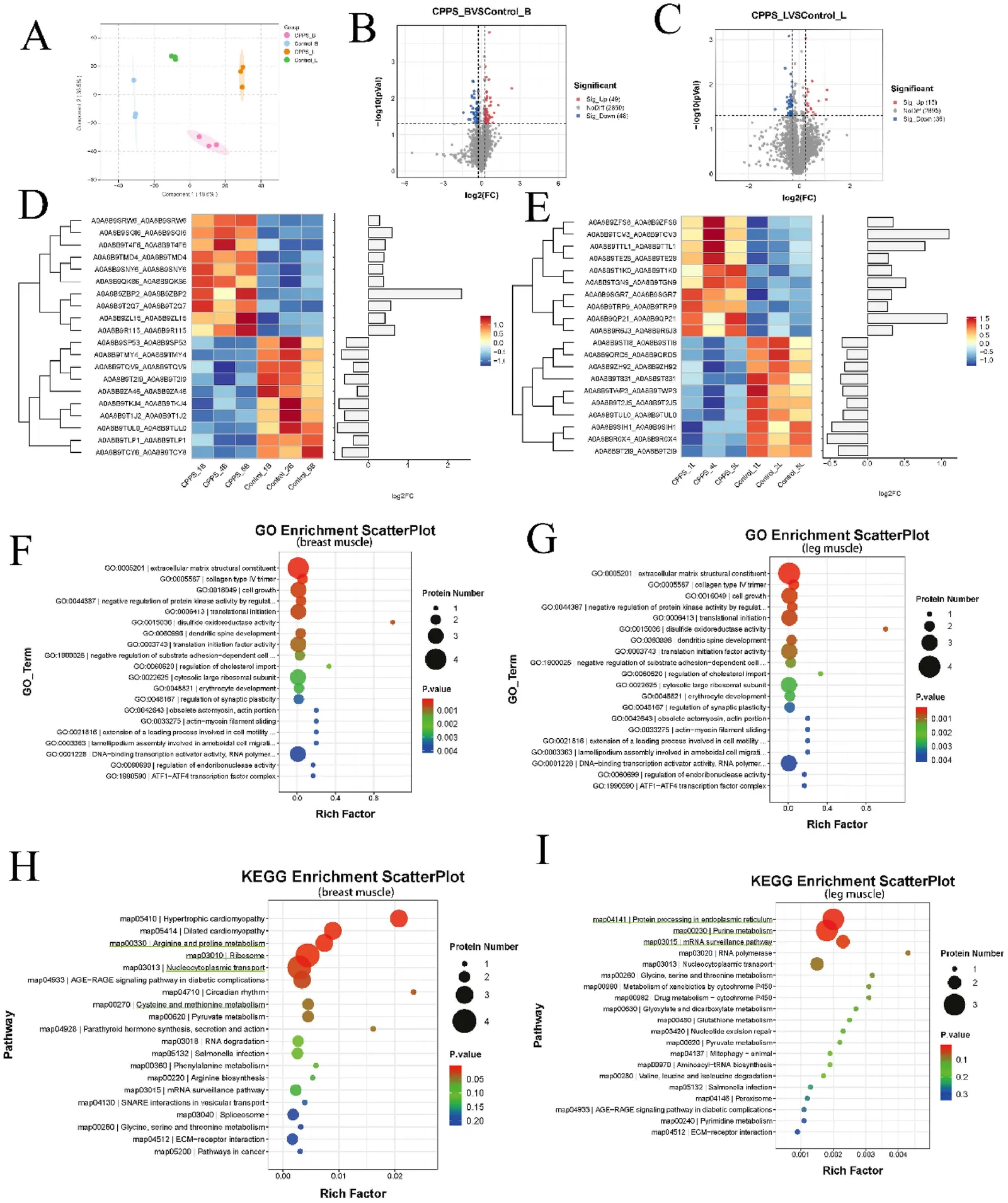
(A) Scatter plot of OPLS-DA model scores for different groups. (B-C) Volcano polt of DEPs, (B) for CPPS_B VS Control_B, (C) for CPPS_L VS Control_L. (D-E) Cluster analysis of DEPs, (D) for CPPS_B VS Control_B, (E) for CPPS_L VS Control_L. (F-G) GO enrichment analysis of DEPs in skeletal muscle. (F) for CPPS_B VS Control_B, (G) for CPPS_L VS Control_L. (H-I) KEGG pathways of DEPs enrichment in skeletal muscle. (H) for CPPS_B VS Control_B, (I) for CPPS_L VS Control_L.

A total of 2947 proteins were identified in breast muscle using LC-MS/MS, the same as the leg muscle. To explore the changes in protein expression across different groups, a multivariate analysis OPLS-DA was conducted, focusing on the variations in differentially expressed proteins (Fig. 2A). The model demonstrated outstanding predictive performance, with R^2^X, R^2^Y, and Q^2^ values of 0.66, 0.99, 0.38, respectively, highlighting its reliable discriminatory and forecasting capabilities. The muscle from different treatment groups were distinctly separated showing no overlap. Interestingly, the samples of CPPS_B, CON_B, CPPS_L, and CON_L were positioned in the fourth, third, first and second quadrants, respectively.

In pairwise comparisons between Control groups and CPPS_B and CPPS_L group, 97 and 52 differentially expressed protein were detected, respectively (Fig. 2B, 2C). The CPPS_B group showed 49 upregulated proteins and 48 downregulated proteins in comparison with Control B samples. Similarly, the upregulated protein counts in CPPS_L were 16 while downregulated proteins were 36. To further illustrate how proteomics distinguished the protein expression across samples, the hierarchical clustering analysis was performed (Fig. 2D, 2E). The results showed differentially proteins can be clearly clustered into two functionally opposite expression clusters: one cluster of proteins is significantly upregulated (highly expressed in red) in the CPPS treatment group, while it is downregulated (lowly expressed in blue) in the Control group; the expression trend of the other cluster of proteins is completely opposite. This suggests that CPPS treatment has a systematic and directional regulatory effect on the proteome, and the expression patterns of the differentially expressed proteins show a high degree of inter-group consistency.

Compared with the CON_B group, the CPPS_B group exhibited significant upregulation of multiple muscle development-associated proteins in breast muscle, including A0A8B9ZBP2 (trans-L-3-hydroxyproline dehydratase), A0A8B9TH91 (actin-related protein 2/3 complex subunit 4), A0A8B9R1M7 (collagen type IV alpha 2 chain), myosin heavy chain (*MyHC*, A0A8B9ZL15), and myomesin 3 (A0A8B9ZBZ5). In contrast, three candidate proteins displayed obvious downregulation, which were A0A8B9TGF0 (sulfotransferase), A0A8B9TC07 (serine incorporator 1), and A0A8B9SJG8 (Ragulator complex protein *LAMTOR1*). For leg muscle, muscle development-related proteins such as A0A8B9TCV3 (phospholamban, *PLN)*, mitofusin 1(*MFN1*, A0A8B9TRP9), titin (A0A8B9ZFJ8), cytokine-like nuclear factor N-PAC (*GLLYR1*, A0A8B9ZFS8), protein transport protein SEC23 (A0A8B9TVT1) and glutathione S-transferase kappa (A0A8B9QP21) were upregulated in CPPS_L group. Meanwhile, RNA polymerase II subunit B (*POLR2B*, A0A8B9QTU2), eukaryotic translation initiation factor 4E (*eIF4E*, A0A8B9QR92), AMP deaminase (*AMPD3*, A0A8B9STI8), RNA-binding protein 38 (*RBM38*, A0A8B9TNN5) and ubiquitinyl hydrolase 1 (A0A8B9TP44) exhibited downregulated expression in CPPS_L group.

### GO and KEGG enrichment analysis of DEPs

The protein functional analysis was conducted using all DEPs based on the GO category enrichment and GO and UniProt databases.

For the CPPS_B VS CON_B, the main GO entries for the enriched differential proteins were extracelluar matrix structural constituent, cell growth, translational initaton, DNA-binding transcription activator activity (*P* < 0.05). KEGG pathway analysis showed that DEPs were mainly arginine and proline metabolism, ribosome, nucleocytoplasmic transport, Cysteine and methionine metabolism (*P* < 0.05). For the CPPS_L VS CON_L, 19 GO terms of were correlated with muscle development, such as skeletal muscle myosin thick filament assembly, muscle myosin complex, skeletal muscle myosin thin filament assembly, muscle alpha-actinin binding were significantly enriched (*P* < 0.05). KEGG pathway analysis showed that DEPs were mainly enriched in protein processing in endoplasmic reticulum, purine metabolism, and mRNA surveillance pathway (*P* < 0.05) (Fig. 2F to I).

### Screening and analysis of DEMs

**Fig. 3:**
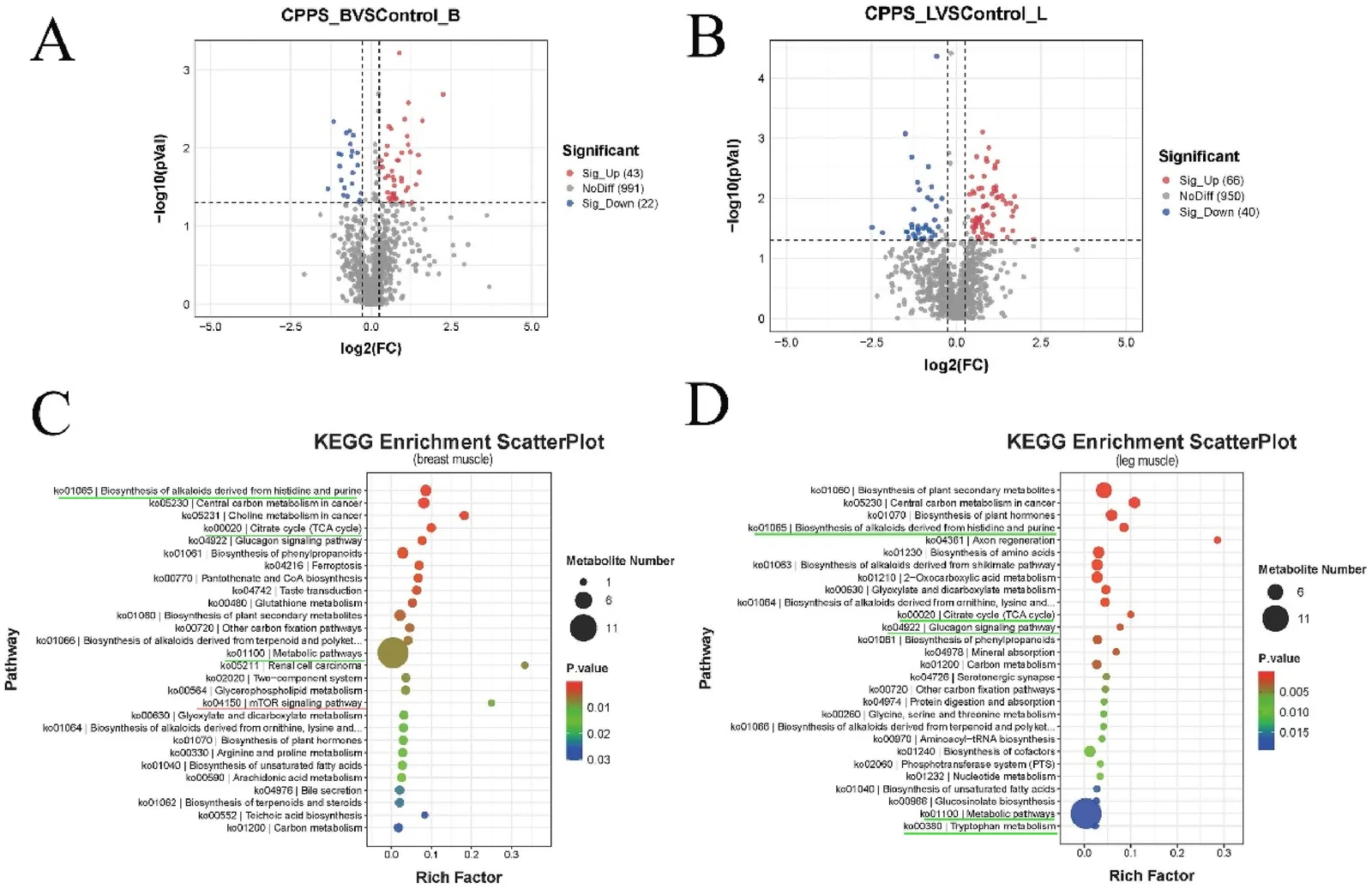
(A-B) Volcano polt of DEMs, (A) for CPPS_B VS Control_B, (B) for CPPS_L VS Control_L. (C-D) KEGG pathways of DEPs enrichment in skeletal muscle. (C) for CPPS_B VS Control_B, (D) for CPPS_L VS Control_L.

In total, 1647 metabolites were identified. The breast muscle in CPPS group showed 43 upregulated metabolites (POS26, NEG17) and 22 downregulated metabolites (POS9, NEG13) in comparison with the CON group (Fig. 3A and B). Similarly, the upregulated metabolites counted in leg muscle were 66, while downregulated metabolites were 40. Among them, 3-methylheptadecanoic acid, Adrenic acid, Dilinoleoylphosphatidylcholine (DLPC), Phosphatidylcholine and Eicosa-11,14,17-trienoic acid are the top five abundant metabolites in breast muscle. Compared with the CON_B, Malic acid, Phosphatidylcholine, Glutathione were significantly upregulated (*P* < 0.05), and diglyceride, Citric acid was significantly downregulated in the CPPS_B group (*P* < 0.05). Of them, Malic acid was the most significantly upregulated metabolite (FC = 1.6, *P* = 0.004) and diglyceride the most significantly downregulated metabolite (FC = −1.1, *P* = 0.004).

Similarly, D-Phenylalanine, Eicosa-11,14,17-trienoic acid, LysoPC, 2-Hydroxyphenethylamine, Ubiquinol-7 are the top five abundant metabolites in leg muscle. Compared with the CON_L, 5-Hydroxy-L-tryptophan, Guanine, Methionine, Flunarizine were significantly upregulated (*P* < 0.05), and Citric acid and Oleic acid were significantly downregulated in the CPPS_L group (*P* < 0.05). Of them, 5-Hydroxy-L-tryptophan was the most significantly upregulated metabolite (FC = 1.51, *P* = 0.002) and Citric acid the most significantly downregulated metabolite (FC = 0.35, *P* = 0.0008).

### KEGG enrichment analysis of DEMs

For the CPPS_B VS CON_B, the KEGG pathway analysis showed that DEMs were mainly enriched in biosynthesis of alkaloids derived from histidine and purine (ko01065), citrate cycle (TCA cycle) (ko00020), Metabolic pathway (ko01100), mTOR signaling pathway(ko04150), glucagon signaling pathway(ko04922) (*P* < 0.05). For the CPPS_L VS CON_L, the KEGG pathway were mainly enriched in Metabolic pathway (ko01100), tryptophan metabolism(ko00380), glucagon signaling pathway(ko04922), biosynthesis of alkaloids derived from histidine and purine (ko01065), citrate cycle (TCA cycle) (ko00020) (*P* < 0.05) (Fig. 3C and D).

### Joint analysis of transcriptomics, preteomics and metablomics

**Fig. 4:**
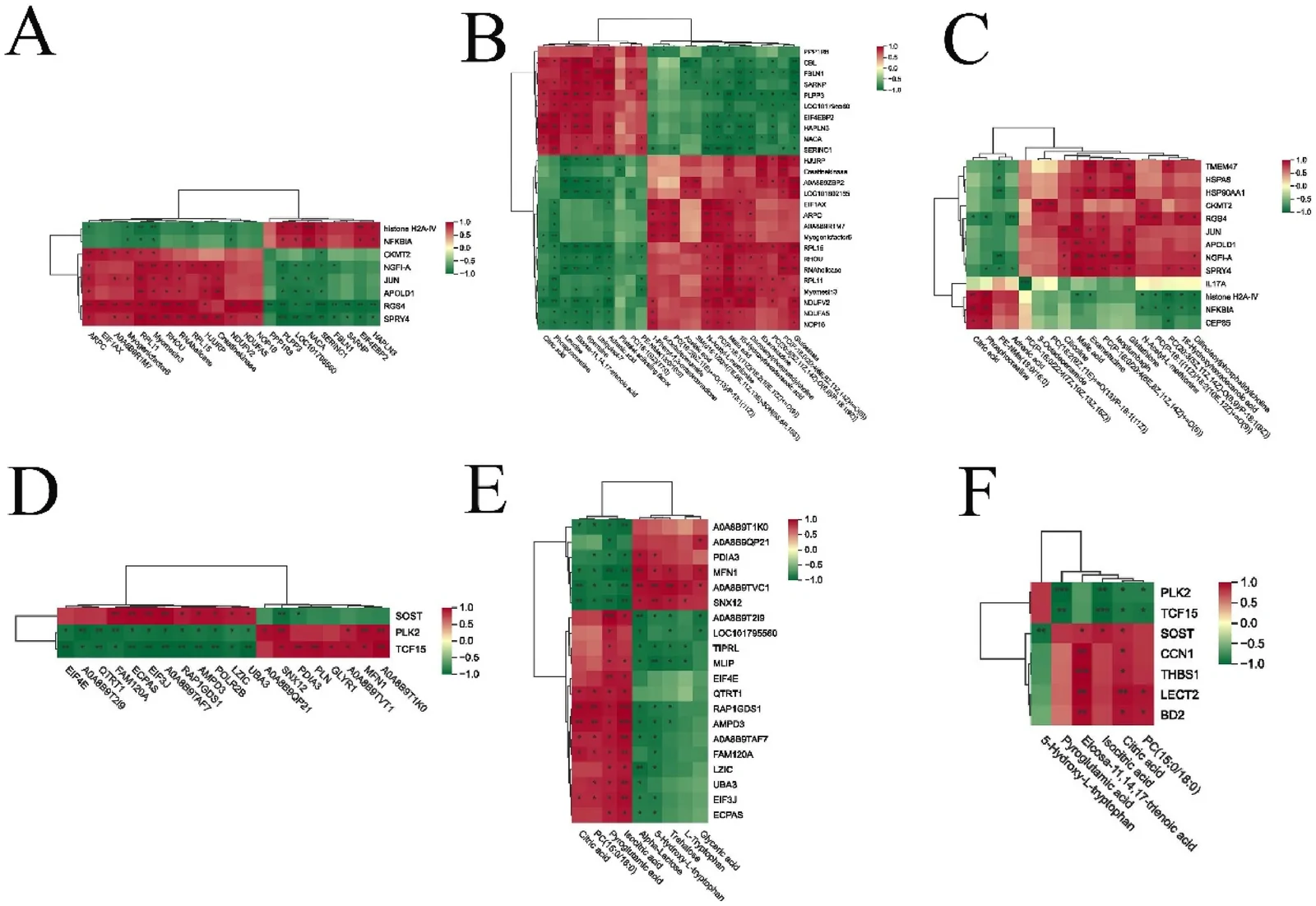
Joint analysis of transcriptomic, proteomic and metabolomic in skeletal muscles. (A) The correlation between DEGs and DEPs in CPPS_B VS Control_B. (B) The correlation between DEPs and DEMs in CPPS_B VS Control_B. (C) The correlation between DEGs and DEMs in CPPS_B VS Control_B. (D) The correlation between DEGs and DEPs in CPPS_L VS Control_L. (E) The correlation between DEPs and DEMs in CPPS_L VS Control_L. (F) The correlation between DEGs and DEMs in CPPS_L VS Control_L.

Based on the comparative analysis results between CPPS_B VS CON_B, 18DEGs, 26 DEPs and 36 DEMs were selected to analysis. In the correlation between DEGs and DEPs (Fig. 4A), the upregulated DEGs, *RGS4, NGFI-A, SPRY4, JUN, APLOLD1* and *CKMT2* were positively correlated with the upregulated DEPs myomesin3 protein (*P* < 0.01 and *P* < 0.05), negatively correlated with histone H2A-IV (*P* < 0.01) and NFKBIA (*P* < 0.05). The upregulated DEPs myogenicfactor 6, creatinekinase addressed the same correlation. In the correlation between DEPs and DEMs_(Fig. 4B), the upregulated DEPs, myomesin3, myogenicfactor 6, creatinekinase and RNAhelicase showed positively correlated glufosinate, dilinoleoylphosphatidylcholine, malic acid, azelaic acid (*P* < 0.01 and *P* < 0.05), and negatively correlated with adrenic acid, spermine, leucine and citric acid (*P* < 0.01 and *P* < 0.05). In the correlation between DEGs and DEMs (Fig. 4C), the upregulated DEGs, *RGS4, NGFI-A, SPRY4, JUN, APLOLD1* and *CKMT2* were positively correlated with glutathione, malic acid, dilinoleoylphosphatidylcholine (*P* < 0.01 and *P* < 0.05), and negatively correlated with adrenic acid and citric acid (*P* < 0.01 and *P* < 0.05). The mTOR signaling pathway and Glycerophospholipid metabolism were the pathways that are collectively enriched with differentially expressed genes, proteins and metabolites (Table 3).

**Table 3:** The list of the DEGs, DEPs and DEMs involved in the shared pathways among transcriptomics, metabolomics and proteomics in breast muscle.

Pathway	Class	Names	CPPS_B VS CON_B		
			log_2_^FC^	*p-*Value	Trend
mTOR-signaling way	Trans	DDIT4 (ENSAPLG00020006884)	−0.43	0.01	down
	Proteo	eIF4E2 (A0A8B9SQK7)	0.60	0.03	up
		A0A8B9SJG8	−0.78	0.02	down
	Metablics	Leucine	0.78	0.05	down
Glycerophospholipid metabolism	Trans	AGPAT5 (ENSAPLG00020012859)	0.46	0.00	up
		LPIN1 (ENSAPLG00020001677)	−1.26	0.01	down
	Proteo	PLPP3 (A0A8B9TMY4)	−0.67	0.0	down
	Metablics	Citicoline	0.51	0.04	up
		PC (36:1)	−0.42	0.02	down

Based on the comparative analysis results between CPPS_L VS CON_L, 15 DEGs, 29 DEPs and 17 DEMs were selected to analysis. In the correlation between DEGs and DEPs (Fig. 4D), the upregulated *TCF15* and *PLK2* were positively correlated with the upregulated DEPs *PLN, MFN1, PDIA3* (*P* < 0.01 and *P* < 0.05), negatively correlated with *AMPD3, EIF4E* (*P* < 0.01 and *P* < 0.05). In the correlation between DEPs and DEMs (Fig. 4E), the upregulated MFN1, A0A8B9TVT1 showed positively correlated L-Tryptophan, 5-Hydroxy-L-tryptophan, Glyceric acid and Alpha-Lactose (*P* < 0.01 and *P* < 0.05), and negatively correlated with Isocitric acid, Pyroglutamic acid and Citric acid (*P* < 0.01 and *P* < 0.05). In the correlation between DEGs and DEMs (Fig. 4F), the upregulated DEGs, *PLK2* and *TCF15* were positively correlated with 5-Hydroxy-L-tryptophan, and negatively correlated with Citric acid, Isocitric acid and Pyroglutamic acid (*P* < 0.01 and *P* < 0.05). The Citric acid was positively correlated with downregulated gene *LECT2* (*P* < 0.01). In the DEGs, DEPs, and DEMs co-enrichment KEGG pathway, none of pathway shared by the three omics, DEGs and DEPs shared 17 pathways, DEPs and DEMs shared 5 pathways, and DEGs and DEMs shared 6 pathways (Table 4).

**Table 4:** The list of the DEGs, DEPs and DEMs involved in the shared pathways among transcriptomics, metabolomics and proteomics in leg muscle.

Compared samples	Kegg Pathway
DEGs VS DEPs (17 pathways)	Calcium signaling pathway, NOD-like receptor signaling pathway, Endocytosis, Ubiquitin mediated proteolysis, Focal adhesion, Salmonella infection, Regulation of actin cytoskeleton, mRNA surveillance pathway, Adrenergic signaling in cardiomyocytes, mTOR signaling pathway, Insulin signaling pathway, RNA polymerase, Mitophagy - animal, Nucleocytoplasmic transport, ECM-receptor interaction, Nucleotide excision repair, Protein processing in endoplasmic reticulum.
DEPs VS DEMs (5 pathways)	mRNA surveillance pathway, Glycine, serine and threonine metabolism, Glyoxylate and dicarboxylate metabolism, Glutathione metabolism, Aminoacyl-tRNA biosynthesis
DEGs VS DEMs (6 pathways)	Metabolic pathways, Glycerolipid metabolism, Glycerophospholipid metabolism, ABC transporters, Biosynthesis of unsaturated fatty acids, Biosynthesis of cofactors

## Discussion

*Codonopsis pilosula polysaccharides* (CPPS) exhibit widely biological activities, including immunomodulatory, antitumor, and antioxidant effects. Also, CPPS plays antiulcer, prebiotics activit.(Zou et al., 2020) In animal husbandry, they are commonly incorporated into feed formulation to enhance the growth performance of livestock and poultry. During the later stage of embryo development, energy is mainly derived from hepatic gluconeogenesis, with the yolk sac providing nutritional substrates for this process until the yolk is resorbed.(Kucharska-Gaca et al., 2017) Insufficient nutrient supply can lead to embryo development obstruction and a decrease in hatching rate. By in ovo feeding (IOF) nutrients into the embryo eggs, the nutritional needs of embryo development can be met, improving the quality of embryo development, and thereby enhancing the hatching efficiency and meat production of poultry.(Das et al., 2021) However, the molecular regulatory mechanism of CPPS on the development of duck embryo skeletal muscle has not been clearly defined. Multi-omics analysis has been widely utilized in the field of animal genetics and breeding. Therefore, the transcriptome, proteome and metabolome, combined with qPCR verification were been used to systematically analyze the molecular changes in duck embryo muscle tissue after IOF of CPPS. The key genes and proteins related metabolites of breast and leg muscle development during embryonic period were explained in this study, and the results providing multi-dimensional evidence for clarifying its mechanism of action.

In the breast muscle, the mTOR signaling pathway and Glycerophospholipid metabolism were the two pathways which shared by differentially expressed genes, proteins and metabolites. The mTOR signaling pathway is the core hub for maintaining skeletal muscle mass. Its precise regulation is of great significance for promoting muscle growth and preventing muscle atrophy.(Yoon, 2017) In addition, glycerophospholipids are the main components cellular membranes, playing a crucial role in maintaining the structure and function of animal cells. (Lordan et al., 2017) Glycerophospholipids also contributed to the cellular metabolism, signaling pathways, and cell type-specific processes like muscle contraction.(Burg et al., 2025)

JUN (ENSAPLG00020016529) (*c-JUN*) was enriched several pathways associated with cell growth and proliferation such as mTOR signaling pathway. Although the FPKM value is relatively low, the unregulated gene *WNT16* (ENSAPLG00020016278) was involved in the same pathway. The upregulated gene *JUN*, a member of Activator protein-1 (*AP-1*) transcription factor complex, mostly act as a positive regulator of cell proliferation.(Shaulian et al., 2001) A research demonstrated that JUN participates in the molecular regulatory network of skeletal muscle growth and development by targeting the activity of the mTORC1 complex (Baraldo et al., 2019). And in Schwann cells, sustained co-activation of *c-JUN* and the mTORC1 signaling pathway has been demonstrated(Della-Flora Nunes et al., 2021). In this study, the IOF of *CPPS* can induce the upregulation of the *JUN* gene expression in the breast muscles of duck embryos, and the results of differential gene function enrichment point to the mTOR signaling pathway.

The downregulated *DDIT4* (ENSAPLG00020006884, DNA damage inducible transcript 4) that was enriched in the mTOR signaling pathway. *DDIT4* has been identified as a key regulator of muscular mass. Mechanistically, *DDIT4* activates *TSC2*, thereby inhibiting the activity of Rheb and preventing the activation of the mTORC1 complex (Inoki et al., 2003; Brugarolas et al., 2004; Bai et al., 2007). Downregulation of *DDIT4* during re-alimentation activates mTORC1 and promotes muscle protein synthesis in beef cattle (Wang et al., 2024). Another research reported that *DDIT4* was upregulated in porcine congenital splay leg (Schumacher et al., 2021). In the present study, downregulated expression of *DDIT4* in CPPS group, suggesting that the mTOR pathway was activated, thereby promoting cellular anabolic metabolism and facilitating cell growth.

Among the differentially expressed proteins, there was an upregulation of *eIF4E2* (Eukaryotic translation initiation factor 4E type 2, *eIF4E2* or *4EHP*, A0A8B9SQK7) and a downregulation of A0A8B9SJG8 enriched in the mTOR signaling pathway. Within the mTOR regulatory network, activated mTORC1 phosphorylates *4EBP1*, leading to its dissociation from eIF4E and subsequent assembly of the *eIF4F* translation initiation complex. Activation of the mTORC1/*eIF4E* pathway promotes *HIF-1α* translation, and elevated *HIF-1α* upregulates multiple glycolytic enzymes, thereby enhancing glycolysis and ATP production (Chen et al., 2023). The cap-binding protein *eIF4E2* is a member of *eIF4E* family, a group key translation initiation factors in eukaryotes (Christie et al., 2021). The *Caenorhabditis elegans* (*Ce*) *4EHP* homolog *IFE-4* (Initiation Factor 4E-4) is expressed in neurons, muscle, spermatheca, and vulva. Although *ife-4* null mutants are viable, they grow slower than wild type and show egg-laying defects and decreased brood size (Dinkova et al., 2005). The *eIF4E* has also been implicated in the regulation of animal growth. Downregulation of *eIF4E* subunit expression may contribute to attenuated muscle hypertrophy in low birth weight neonatal pigs (El-Kadi et al., 2018). However, a downregulated protein A0A8B9SJG8 (Ragulator complex protein LAMTOR1) acts as the core subunit of Ragulator and is responsible for recruiting mTORC1 to the lysosomal surface and activate mTORC1 (Bar-Peled et al., 2012). Downregulation of LAMTOR1 indicates that CPPS restricts excessive assembly and persistent activation of mTORC1 on the lysosomal surface, likely serving as a physiological negative feedback response to avoid hyperproliferation of muscle cells and metabolic disorders. Furthermore, the downregulated differential metabolites leucine also enriched in mTOR signaling pathway. Leucine is necessary for mTORC1 activation (Drummond et al., 2009). However, the decrease in intracellular free leucine indicates that the amino acids are being used for muscle protein synthesis, rather than the inactivation of mTORC1 (Suryawan et al., 2012).

Thus, we assume that the multi-omics results demonstrate that CPPS acts not via simple and sustained mTORC1 activation, but through a precise “moderate activation - negative feedback balance” mode to safely and effectively promote duck embryonic breast muscle development.

Another pathway that was co-enriched in all three omics was the glycerophospholipid metabolism pathway. Glycerophospholipid metabolic pathway is the material basis and signal hub for muscle development. In a lipidome study during the growth stage of the breast muscle of geese, glycerides and glycerophospholipids were the predominant components in the IMF of goose meat, and glycerophospholipid metabolism was the pathways most significantly associated with lipid changes during goose growth (Cao et al., 2024).

In the breast muscle after IOV of CPPS, the differentially upregulated 1-acylglycerol-3-phosphate O-acyltransferase 5 (*AGPAT5*, ENSAPLG00020012859) gene, the downregulated *LPIN1* (*Lipin 1*, or phosphatidic acid phosphatase, *PAP1*, ENSAPLG00020001677) gene, the downregulated *PLPP3* (Phospholipid phosphatase 3, A0A8B9TMY4) protein, and the upregulated metabolite citicoline and the downregulated metabolite Phosphatidylcholine (PC) (36:1) were all co-enriched in the glycerophospholipid metabolism pathway. *AGPAT5* is a enzyme that catalyze the conversion of lysophosphatidic acid to phosphatidic acid(*PA*), which is a precursor of triacylglycerol (Takeuchi et al., 2009). *AGPAT5* has been shown to play an import role in meat weight in cattle and a potential genetic marker for excellent pork quality (Edea et al., 2020; Molinero et al., 2022). *LPIN1* catalyzes the conversion of *PA* to diacylglycerol (DAG) (Takeuchi et al., 2009). The downregulation of *LPIN1* indicates that a key step in the glycerophospholipid metabolic pathway has been inhibited. *PLPP3* has the ability to dephosphorylate *PA* (Carman et al., 2011)*.* Downregulated *PLPP3* protein indicated that reduced the ability. These three factors working together led to the accumulation of *PA*. It has been reported that oral or intracellular *PA* significantly activates mTOR and significantly improved responses in skeletal muscle hypertrophy (Joy et al., 2014). Citicoline is an essential intermediate in the biosynthetic pathway of the structural phospholipids of cell membranes (Secades et al., 2022). The upregulation of citicoline indicated that CPPS directly promotes the synthesis of phosphatidylcholine, providing raw materials for the expansion of muscle cell membranes. Phosphatidylcholine (*PC*) (36:1) decreased, indicating that the phosphatidylcholine of a specific molecular type decreased, suggesting that the composition of glycerophospholipids underwent adaptive remodeling to optimize membrane fluidity and signal function, rather than a general decrease in *PC*. In a study on human breast cancer, it was reported that only *PC* (36:1) was significantly enriched in breast cancer tissues, which was positively correlated with the high expression of *SCD1* (stearoyl-CoA desaturase 1). Our research has consistently shown that after CPPS injection, downregulation of *PC* (36:1) and *SCD1* gene were observed in the breast muscle.

These results suggested that CPPS can reshape the phospholipid composition of muscle cell membranes and lipid metabolic homeostasis, thereby participating in the regulation of skeletal muscle development. The synergistic effect of glycerophospholipid metabolism and the mTOR pathway is an important metabolic mechanism by which CPPS promote the growth and development of duck breast muscles.

In addition to the two common enriched pathways, other critical changes and biological traits were found in the differential genes, proteins, and metabolites of breast muscle. Among the upregulated genes, regulator of G protein signaling 4 (*RGS4*, ENSAPLG00020014659) and creatine kinase mitochondrial 2 (*CKMT2*, ENSAPLG00020008721) are related to energy metabolism but were not identified in the KEGG enrichment analysis. The regulation of G protein signaling by *RGS4* may indirectly affect the activity of key enzymes in energy metabolism pathways such as glycolysis and oxidative phosphorylation. Studies have reported that the activity of the neural system's mTOR is specifically inhibited due to the absence of *RGS4* (Mikdache et al., 2021). *CKMT2* has been confirmed as a key regulatory factor for skeletal muscle growth by regulating the proliferation and differentiation of myoblasts (Xie et al., 2025). Among the down-regulated genes, histone H2A-IV was enriched in the Necroptosis pathway, which indicated that CPPS may regulate histone modification to inhibit programmed necrosis in skeletal muscle cells, reduce abnormal cell death, thereby protecting muscle cells and facilitating the growth and development of the breast muscles. The downregulation of the gene *NFKBIA* (ENSAPLG00020000650), a gene of canonical *NF-κB* activation pathway, have been proposed as risk *loci* for many immune-mediated diseases, suggested that the CPPS may activate the NF-κB signaling pathway (Zhang et al., 2011). *SCD1* is rate-limiting enzyme that catalyzes the synthesis of monounsaturated fatty acids (Jiang et al., 2008; Stamatikos et al., 2013). The decrease in its expression indicates that CPPS s can regulate lipid metabolism and membrane phospholipid remodeling by inhibiting *SCD1*-mediated monounsaturated fatty acid synthesis, thereby affecting muscle cell growth, membrane stability, and energy metabolism. Dickkopf-related protein 2 (*DKK2*) is a typical inhibitor of the Wnt signaling pathway (Song et al., 2025). The downregulation of *DKK2* may indicate that the Wnt signaling pathway may be activated.

Furthermore, at the protein level, the upregulation of myomesin 3 (A0A8B9ZBZ5), myogenic factor 6 (A0A8B9SGC1), myosin heavy chain and other myogenic-related proteins (A0A8B9ZL15, *MyHC*), together with elevated creatine kinase, directly indicated that mTOR signaling facilitates muscle protein synthesis and promotes myofiber development. At the metabolic level, increased level of malic acid, phosphatidylcholine, and glutathione, coupled with reduced triglycerides and citric acid, optimize muscle energy supply and maintain oxidative stress homeostasis, thereby providing essential metabolic support for the activation of the mTOR signaling pathway. Collectively, these findings demonstrate that CPPS could regulate the mTOR signaling pathway, glycerophospholipid metabolism, as well as the Wnt signaling pathway, necroptosis pathway, and other related pathways in a multi-dimensional manner. These coordinated regulations synergistically promote duck breast muscle fiber development, enhance energy metabolism, alleviate stress-induced injury, and ultimately improve skeletal muscle growth potential and meat quality in ducks.

Although no pathway was jointly enriched across all three omics levels in leg muscle, a considerable number of pathways were overlapped between two-omics comparisons. Specifically, 17 pathways were shared between differentially expressed genes (DEGs) and differentially expressed proteins (DEPs), 5 pathways between DEPs and differentially expressed metabolites (DEMs), and 6 pathways between DEGs and DEMs.

In the DEGs of leg muscle, the upregulated gene transcription factor 15 (*TCF15*, ENSAPLG00020006012). *TCF15* (formerly named paraxis) is a transcription factor that falls within the basic helix-loop-helix (*bHLH*) family, which associated with muscle differentiation and development (Mohammadabadi et al., 2021; Limbach et al., 2022). The upregulation of *TCF15* suggests that CPPS could improve duck embryonic skeletal muscle development by facilitating myogenic cell differentiation and early myogenesis. Besides, the upregulated polo-like kinases (*PLK2*, also known as *SNK*), an evolutionarily conserved serine/threonine kinase, functions as a critical regulator of S-phase progression (de Cárcer et al., 2011). Previous studies have demonstrated that *PLK2* serves as a critical negative regulator of myocardial fibrosis(Künzel et al., 2021). Moreover, CPPS downregulated the expression of leukocyte cell-derived chemotaxin 2 (*LECT2*) in leg muscle, a well-documented pro-fibrotic factor in the liver and kidney (Xu et al., 2019; Hu et al., 2025). Based on these findings, we speculate that CPPS may contribute to the attenuation of muscle fibrosis and help maintain tissue homeostasis in duck embryonic skeletal muscle.

In the DEPs of leg muscles, CPPS upregulated the phosphorus receptor protein (A0A8B9TCV3, *PLN)*, mitofusin 1(A0A8B9TRP9, *MFN1*), titin(A0A8B9ZFJ8), and downregulated *POLR2B* (DNA-directed RNA polymerase subunit beta, A0A8B9QTU2), Eukaryotic translation initiation factor 4E (eIF4E, A0A8B9QR92), AMP deaminase (*AMPD3*, A0A8B9STI8). *PLN* maintains cytoplasmic calcium homeostasis by regulating calcium uptake in the sarcoplasmic reticulum and is a key upstream molecular basis for the activation of the *Ca2^+^ MKII* pathway and the normal transduction of downstream signals (Mattiazzi et al., 2014). Calcium is delivered to mitochondria by *MAM* and activates *PGC-1α*, which is directly transcribed to promote the expression of *MFN1* (Chen et al., 2022; Balderas et al., 2024). *MFN1*, a GTPase located on the outer membrane of mitochondria, mediate mitochondrial fusion and maintain the reticular structure and normal shape of mitochondria (Chen et al., 2003). The absence of *MFN1* can lead to severe muscle atrophy (Chen et al., 2010). Moreover, the titin, as the molecular scale of muscle segments, provides an assembly scaffold for actin and myosin, and its expression level directly determines the length and quantity of muscle fibers (Granzier et al., 2005). *PLN, MFN1* and *Titin* jointly promote the proliferation of muscle cells and the development of muscle fibers. *AMPD3* is a cytoplasmic enzyme involved in adenine nucleotide metabolism, capable of catalyzing irreversible amination of *AMP* (*AMP→IMP+NH₃*), and its high expression is associated with muscle atrophy (Miller et al., 2021). In present study, CPPS downregulated *AMPD3* protein in leg muscles, suggesting that it can reduce *AMP* degradation, maintain nucleotide pool homeostasis, improve energy metabolism, and thereby delay muscle atrophy and protect muscle fiber function.

In the DEMs, CPPS upregulated the 5-Hydroxy-L-tryptophan (5-HTP), guanine, flunarizine, and downregulated citric acid and oleic acid in leg muscle. The 5-HTP is a metabolite that shows a significantly differentially elevated level, which is the precursor for the synthesis of serotonin (5-HT). Previous studies have demonstrated that 5-HT participates in the maintenance of skeletal muscle metabolic homeostasis by regulating energy metabolism, mitochondrial function, and protein synthesis turnover, and is closely associated with muscle weight and muscle fiber development (Musumeci et al., 2015). Guanine as the core product of purine is involved in the regulation of DNA replication and RNA transcription, which is essential for myoblast proliferation and muscle growth (Duperray et al., 2023). Methionine, as a key regulatory factor for the synthetic metabolism of muscle cells, contributes to protein synthesis and mTOR signaling activation, thereby promoting skeletal muscle hypertrophy and increasing muscle weight (Zhou et al., 2016). Studies have reported that flunarizine can protect the integrity of skeletal muscle fibers in spinal muscular atrophy (SMA) model mice by inhibiting calcium overload and stabilizing mitochondrial membrane potential, reducing muscle cell apoptosis and atrophy; at the same time, it can improve mitochondrial respiratory function and ATP production, and enhance the efficiency of muscle energy metabolism (Sapaly et al., 2018). Inhibition of the mitochondrial citrate transporter *SLC25A1* decreases cytoplasmic citrate levels, alleviates high-fat diet-induced obesity, and normalizes the expression of lipolysis-related genes (Wang et al., 2024). Moreover, *ACLY* inhibition markedly reduces malonyl-CoA levels and blocks fatty acid synthesis (Li et al., 2018). A study has pointed out that after oleic acid treatment of bovine skeletal muscle satellite cells, the factors related to muscle production (*Pax3* and *MyoD*) were significantly downregulated, while the factors related to lipid generation (*C/EBP-β* and *PPARγ*) were significantly upregulated, promote cell lipid transdifferentiation. Thus, our results suggest downregulation of citric acid and oleic acid, indicated that CPPS may reduce fat deposition in leg muscles and prevent excessive accumulation of fat within the muscles.

A total of 10 randomly selected differentially expressed genes (DEGs) were validated by qRT-PCR, including *CKMT2, RGS4*, histone H2A-IV, *NFKBIA, DKK2, DDIT4*, and *SCD1* in breast muscle, as well as *TCIF15, LETC2*, and *MRP-126* in leg muscle. These validation results indicate that the DEGs identified by high-throughput RNA-seq are highly reliable and reproducible.

Multi-omics analysis in the breast muscle revealed the mTOR signaling pathway and glycerophospholipid metabolism were identified as key pathways jointly enriched by DEGs, DEPs, and DEMs. These pathways are critically involved in protein synthesis, energy metabolism, mitochondrial function, and muscle fiber development. Nevertheless, although the multi-omics analysis in leg muscle did not identify commonly enriched KEGG pathways, the correlated profiles of differential genes, proteins, and metabolite suggest that CPPS may promote leg muscle development and enhance muscle quality by upregulating the expression of myogenic genes and translation initiation factors to provide a stable molecular environment and sufficient energy supply for muscle protein synthesis, while inhibiting adverse processes including muscle cell fibrosis, muscle atrophy, and excessive intramuscular fat deposition.

The present study clarified the regulatory mechanism of CPPS on embryonic muscle development in ducks from a multi-omics perspective. Meanwhile, the health-promoting effects of CPPS in livestock and poultry production have also been widely documented and verified by numerous studies. Previous studies have primarily focused on the antioxidant, anti-stress, anti-inflammatory, immunoregulatory, and therapeutic effects of CPPS in livestock and poultry, whereas studies investigating its impact on skeletal muscle development remain limited. Previous studies have demonstrated that CPP significantly increase muscle glycogen and liver glycogen contents in mice, reduce lactate and urea nitrogen levels, and exert anti-fatigue and muscle-protective effects by optimizing systemic energy metabolism(Xie et al., 2020). Recent studies have further indicated that CPPS improves mitochondrial respiratory function, enhances ATP synthesis efficiency, and regulates the tricarboxylic acid cycle and nucleotide metabolism processes, thereby improving muscle energy metabolism and delaying muscle atrophy (Li et al., 2025; Yao et al., 2025). In addition, *Codonopsis lanceolata* (CL) can ameliorate sarcopenic obesity by restoring the PI3K/Akt signaling pathway and modulating lipid metabolism in skeletal muscle (Han et al., 2025).

The limitation of our study are the key metabolic pathways were identified and analyzed only based on multi-omics data, lacking in vitro cellular validation and mechanistic verification, and the precise regulatory effects of these pathways have not been confirmed by functional experiments. These aspects will be the focus of our future research. In addition, this study only focused on the embryonic stage of ducks. In future work, the experimental period could be extended to the marketing stage to systematically investigate the long-term effects of embryonic administration of CPPS on skeletal muscle yield, meat quality traits, egg quality and other economic characteristics at different growth stages of ducks, so as to provide a more comprehensive scientific basis for its practical application in production. 

Therefore, our multi-omics integration analysis revealed that CPPS promotes breast muscle development in duck embryos mainly through the mTOR signaling pathway and glycerophospholipid metabolism as the core regulatory pathways. Although no commonly enriched KEGG pathway was detected for CPPS-mediated regulation in leg muscle, the overall changes in differential molecules still support the beneficial effects of CPPS on leg muscle development. Collectively, CPPS effectively improves muscle energy metabolism, alleviates metabolic stress, facilitates myofiber growth, and optimizes meat quality. This study provides multi-omics evidence for clarifying the molecular mechanism underlying the regulation of embryonic muscle development by *Codonopsis pilosula polysaccharides*, and establishes a theoretical foundation for the application of CPPS as a nutritional enhancer in poultry embryos, as well as for improving muscle development and production performance in waterfowl.

## Funding sources

This work was supported by National Natural Science Foundation of China (Grant No.31960682).

## Author Contribution

Ai Liu performed experiments, collected data, analyzed and interpreted results, and drafted the manuscript. Yifu Rao contributed to experiment preparation and sample collection. Yong Yue participated in data analysis. Jinjin Zhu, Jiying Wen and Yongcai Zhu assisted in carrying out experiments. Biqiong Yao helped prepare experimental materials. Surintorn Boonanuntan provided critical comments. Shenglin Yang secured research funding and critically revised the manuscript.

## Disclosures

The authors confirm that they have no financial or non-financial conflicts of interest with any individuals or organizations that may affect the objectivity of this research.
